# Using Chronopotentiometry to Better Characterize the Charge Injection Mechanisms of Platinum Electrodes Used in Bionic Devices

**DOI:** 10.3389/fnins.2019.00380

**Published:** 2019-04-24

**Authors:** Alexander R. Harris, Carrie Newbold, Paul Carter, Robert Cowan, Gordon G. Wallace

**Affiliations:** ^1^ARC Centre of Excellence for Electromaterials Science, Intelligent Polymer Research Institute, University of Wollongong, Wollongong, NSW, Australia; ^2^The HEARing CRC, University of Melbourne, Melbourne, VIC, Australia; ^3^Department of Audiology and Speech Pathology, University of Melbourne, Melbourne, VIC, Australia; ^4^Cochlear, Ltd., Macquarie University, Sydney, NSW, Australia

**Keywords:** platinum, chronopotentiometry, cochlear implant, impedance test, charge transfer mechanism

## Abstract

The safe charge injection capacity and charge density of neural stimulating electrodes is based on empirical evidence obtained from stimulating feline cortices. Stimulation induced tissue damage may be caused by electrochemical or biological mechanisms. Separating these mechanisms requires greater understanding of charge transfer at the electrode-tissue interface. Clinical devices typically use a biphasic waveform with controlled current. Therefore, the charge injection mechanism and charge injection capacity of platinum was assessed on a commercial potentiostat by chronopotentiometry (controlled current stimulation). Platinum is a non-ideal electrode, charge injection by chronopotentiometry can be passed via capacitive and Faradaic mechanisms. Electrodes were tested under a variety of conditions to assess the impact on charge injection capacity. The change in electrode potential (charge injection capacity) was affected by applied charge density, pulse length, pulse polarity, electrode size, polishing method, electrolyte composition, and oxygen concentration. The safe charge injection capacity and charge density could be increased by changing the electrode-solution composition and stimulation parameters. However, certain conditions (e.g., acid polished electrodes) allowed the electrode to exceed the water electrolysis potential despite the stimulation protocol being deemed safe according to the Shannon plot. Multiple current pulses led to a shift or ratcheting in electrode potential due to changes in the electrode-solution composition. An accurate measure of safe charge injection capacity and charge density of an implantable electrode can only be obtained from suitable conditions (an appropriately degassed electrolyte and clinically relevant electrode structure). Cyclic voltammetric measurement of charge storage capacity can be performed on implantable electrodes, but will not provide information on electrode stability to multiple chronopotentiometric pulses. In contrast, chronopotentiometry will provide details on electrode stability, but the minimum time resolution of typical commercial potentiostats (ms range) is greater than used in a clinical stimulator (μs range) so that extrapolation to short stimulation pulses is required. Finally, an impedance test is typically used to assess clinical electrode performance. The impedance test is also based on a biphasic chronopotentiometic waveform where the measured potential is used to calculate an impedance value. Here it is shown that the measured potential is a function of many parameters (solution composition, electrode area, and surface composition). Subsequently, impedance test results allow electrode comparison and to indicate electrode failure, but use of Ohm’s law to calculate an impedance value is not valid.

## Introduction

Electrodes are used to stimulate excitable cells in cell culture and in the body (Normann and Fernandez, 2016). Electrical stimulation in humans has been used to: provide sensory input, as in the cochlear implant or bionic eye (Christie et al., 2016; Dhanasingh and Jolly, 2017); control body function, such as deep brain stimulation to reduce body tremor (McIntyre et al., 2015); or to affect or influence behavior, including treatments for depression (Kennedy et al., 2011). Sufficient charge must be ejected from the electrode into tissue to induce the desired clinical outcomes without damaging the electrode or the surrounding tissue. Some issues associated with electrical stimulation include off-target stimulation (Lukins et al., 2014), glial cell or fibrous tissue encapsulation (Niparko et al., 1989) and electrode corrosion (Clark et al., 1983; Robblee et al., 1983). As a result of these factors, implants can induce cell death, show an increase in power usage over time, or fail completely.

The safe stimulation parameters of an electrode are currently defined by the Shannon plot which relates the charge density and charge per phase of an electrical pulse (Shannon, 1992). This is based on electrical stimulation of a feline cortex using platinum or tantalum pentoxide electrodes with a limited stimulation protocol. Clinical electrodes use a variety of stimulation waveforms, typically involving a biphasic reduction and oxidation pulse of varying length, amplitude, repetition rate, and interphase gap that may not be covered by the Shannon plot (McCreery et al., 1992). More recent work has also indicated the Shannon plot may not be valid when applied to microelectrodes (Cogan et al., 2016). The validity of the Shannon plot for different electrode materials and in different tissue also needs to be addressed in more detail. These limitations raise questions about what chemical and biological mechanisms determine safe charge densities, and how safe charge injection capacity and density should be measured and reported.

It is assumed that increasing the charge injection capacity of an electrode will increase its safe charge density by preventing unwanted reactions occurring at the electrode-tissue interface. Significant effort has been spent developing novel electrode materials and geometries to increase the charge injection capacity of an electrode (Wang et al., 2017; Harris and Wallace, 2018). The charge injection capacity of an electrode is typically assessed by electrochemical methods, but with limited theoretical basis, the methods used differ across laboratories and lack sufficient controls. This has resulted in poor correlation between *in vitro* and *in vivo* performance (Prasad and Sanchez, 2012). Subsequently, new materials and geometries demonstrating high charge injection capacities have had poor translation to clinical use. A stronger theoretical understanding of how charge transfer occurs at the electrode-tissue interface is needed to define how electrodes should be tested and what are safe charge densities across different electrode and tissue parameters (Kumsa et al., 2016a). This will reduce concerns of platinum dissolution, changes in pH and generation of gas occurring at the electrode surface (Robblee et al., 1983; Agnew et al., 1986; Merrill et al., 2005). It will also help translate the safe stimulation parameters measured in feline cortex to other devices and tissues such as cochlear implants and the bionic eye.

The safe potential window and charge storage capacity of implantable electrodes are often assessed by cyclic voltammetry. However, stimulation of excitable cells with an electrode requires altering the potential across a cell membrane and is usually achieved with electrical pulsing rather than a potential sweep. When performing electrical pulsing, either the potential or current can be controlled while the other parameter varies with time. The response of a platinum electrode under various biologically relevant conditions during controlled potential pulsing (chronoamperometry) was recently investigated (Harris et al., 2018a). Using chronoamperometric pulsing, the electric field decreases rapidly with distance from the electrode; so the charge delivered may not induce sufficient change in membrane potential to excite a cell. The amount of charge delivery also depends on conditions and decreases rapidly with time. In bionics applications, the electrode is normally used in controlled current mode (chronopotentiometry). For instance, modern cochlear implants use a biphasic waveform composed of a μs timescale chronopotentiometic reduction pulse followed by an interphase gap and oxidation pulse. Using chronopotentiometic pulsing, charge delivery will be constant, but the electrode potential is uncontrolled. Furthermore, the *in vivo* performance of the electrode/tissue interface is often assessed by an impedance test (Newbold et al., 2004, 2010, 2011). This is not to be confused with electrical impedance spectroscopy (EIS), or the unit of impedance (*Z*). The impedance test also applies a biphasic chronopotentiometic pulse through each electrode measuring the change in potential, which is then used to calculate an impedance value using Ohm’s law.

Electrodes used for neural stimulation are often referred to as capacitive (ideally polarizable) or Faradaic (ideally non-polarizable) (Cogan, 2008). When analyzing a chronopotentiometic measurement, an ideally polarizable electrode only passes current (*i*_c_) through charging of the double-layer capacitance per unit area (*C*_d_) across the electrode surface with area (*A*) according to

(1) $$i_{\text{c}}=-AC_{\text{d}}(dE/dt)$$

supplying capacitance current requires a constantly changing potential (*E*) over time (*t*). An ideally non-polarizable electrode only delivers charge via a Faradaic reaction, in which case the electrode potential would not change with an applied current.

In practice, no electrode is ideal, with current supplied by capacitance and Faradaic reactions at different potentials and mass transport affects the Faradaic current. When mass transport is present, the current of a Faradaic reaction (*i*_f_) is controlled by the flux conditions

(2) $$i_{f}=nFAD{\left(\frac{\partial C}{\partial x}\right)}_{x=0}$$

where *n* is the number of electrons transferred, *F* is Faraday’s constant, *D* is the diffusion coefficient, *C* is the concentration and *x* is the distance from the electrode (for the simple one dimensional case). These conditions are further affected by electrode geometry and the reversibility of the Faradaic reactions. The total current at a non-ideal electrode, *i* = *i*_c_ + *i*_f_, therefore has varying proportions of capacitance and Faradaic current over time (De Vries, 1968).

To ensure safe stimulation of cells and to prevent degradation of the electrode, the charge delivery mechanisms must not be damaging or create toxic species. The electrode potential must be kept below levels that would corrode the electrode or cause water electrolysis. However, the composition of the electrode-tissue interface is very complex. An implanted platinum electrode is usually multicrystalline, can have varying amounts of oxide present, and the surrounding fluid is composed of various ions, biomolecules, and cells. Therefore, multiple mechanisms can be involved in charge transfer at the electrode-tissue interface, and the safe charge injection capacity will depend on the local conditions. A systematic analysis of changes in stimulation waveform and electrode-solution composition on the chronopotentiometric response of a platinum electrode under well-controlled conditions will help determine their potential impact on charge delivery mechanisms occurring during *in vivo* electrical stimulation.

To better understand the charge delivery mechanisms occurring during stimulation of excitable cells and the most appropriate way of testing implantable electrodes, this study investigated the use of chronopotentiometry to measure the performance of platinum electrodes under a variety of conditions. The impact of solution composition, oxygen concentration, electrode size, electrode polishing method, and applied potential on the chronopotentiometric response of platinum is reported. The possible charge transfer mechanisms occurring under these conditions and the implications for *in vivo* performance are discussed. The relationship between cyclic voltammetry, chronoamperometry, and chronopotentiometry using commercial potentiostats as methods for investigating implantable electrodes and the implication on impedance testing are explained.

## Materials and Methods

### Chronopotentiometric Waveform and Analysis

Chronopotentiometic experiments were performed with repetitive oxidation and reduction pulses of opposing polarity but equal time and magnitude. This is equivalent to a biphasic pulse with no interphase gap. To investigate the stability of the electrode to repeated pulsing, eight reduction/oxidation (four biphasic) pulses were applied. The charge densities chosen were 3 and 10 μC cm^-2^, as they are the typical minimum and maximum limits used by modern cochlear implants. The current pulses in a cochlear implant are typically applied for 25 μs, however the shortest pulse achievable on a CH Instruments potentiostat is 5 ms. A 5 ms pulse (10 ms biphasic pulse) is equivalent to 100 pulses per second; doubling the pulse length to 10 ms is equivalent to 50 pulses per second. The applied charge (*Q*) was calculated by multiplying the charge density by the nominal electrode area (*A*); the current (*i*) for the 5 ms time pulse was then calculated from the total charge passed and the time (*t*), *i* = *Q*/*t*. For instance, achieving a current density of 10 μC cm^-2^ on a 600 μm diameter electrode with a 5 ms pulse, required an applied current of 5.65 μA, resulting in 28.3 nC phase^-1^, this charge density and charge per phase is considered safe according to the Shannon plot (Cogan et al., 2016). The current was adjusted to ensure the same charge density of 10 μC cm^-2^ was applied for each electrode size. By definition, the larger the change in potential measured during the chronopotentiometric pulse, the smaller is the electrodes charge injection capacity. The stability of the electrode to multiple pulsing was assessed by measuring the change in potential from the end of the second pulse to the end of the last pulse (cumulative six pulses).

### Chemicals

Phosphate-buffered saline (PBS: 154 mM NaCl, 10 mM phosphate buffer, pH 7.4), sodium chloride, potassium chloride, sodium bicarbonate, calcium chloride, D-glucose (Sigma-Aldrich), magnesium chloride hexahydrate (Scharlau), monosodium phosphate (Biochemicals) and 98% sulfuric acid (RCI Labscan), were used as received. An artificial perilymph contained 125 mM NaCl, 3.5 mM KCl, 25 mM NaHCO_3_, 1.2 mM MgCl_2_, 1.3 mM CaCl_2_, 0.75 mM NaH_2_PO_4_, and 5 mM glucose (Salt et al., 2003). Unless indicated, test solutions were degassed with nitrogen for at least 10 min.

### Electrodes

Electrodes were 2 mm, 0.6 mm, or 25 μm diameter platinum disks (CH Instruments) or a cochlear implant with 22 half band, 0.3 mm^2^ nominal area platinum electrodes (donated by Cochlear, Ltd.). One electrode of each type was tested. The electrodes were freshly polished before every experiment ensuring reproducible starting conditions. Disk electrodes were polished with 0.3 μm alumina slurry on Microcloth polishing cloth (Buehler), rinsed in deionised water and gently dried (Kimwipe) before use; the cochlear implant was not mechanically polished before use and had not been used for any *in vivo* studies. Acid polishing was achieved by cycling the electrode potential from 1.2 to -0.2 V at 50 mV s^-1^ for 50 cycles in 0.5 M H_2_SO_4_. Electrodes were tested in a 3-electrode configuration on a CHI660E potentiostat (CH Instruments) using a Ag/AgCl (3 M KCl) as reference electrode and Pt wire as counter electrode. The electrodes were connected to the potentiostat via alligator clips and placed into a beaker of solution.

## Results

### Varying Chronopotentiometric Waveform

**FIGURE 1:**
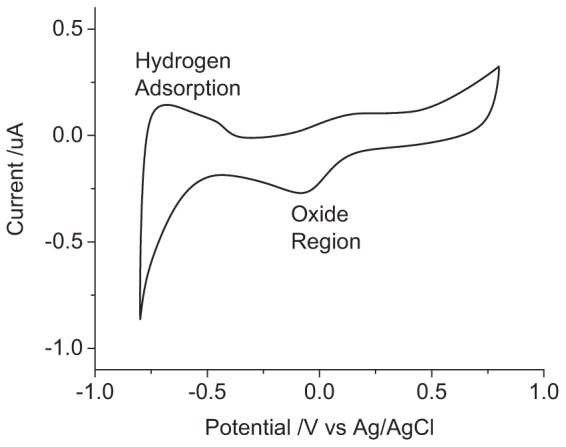
Second cycle of a cyclic voltammogram in degassed 0.1 M NaCl of a mechanically polished 0.6 mm diameter platinum electrode at 100 mV s^-1^.

The voltammetric reduction sweep of a platinum electrode in 0.1 M NaCl displays a peak at -85 mV from reduction of platinum oxide and dissolved oxygen and increasing reduction current below -470 mV from hydrogen adsorption (Figure 1). On the oxidation sweep, broad peaks are seen around -700 to -400 mV from hydrogen stripping and above 0 V from platinum oxide formation. At potentials above and below the potential window of 800 to -800 mV, water oxidation, and reduction can occur.

**FIGURE 2:**
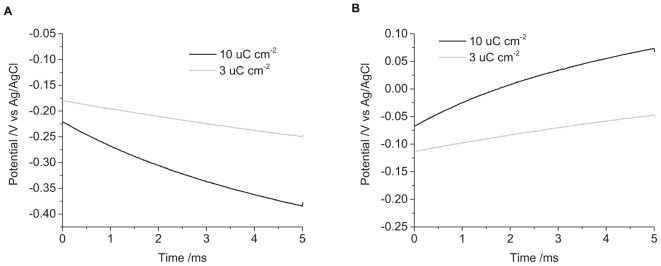
Chronopotentiometric curves of a mechanically polished 0.6 mm diameter platinum electrode in degassed 0.1 M NaCl. **(A)** cathodic pulse, **(B)** anodic pulse. Black curve – current density of 10 μC cm^-2^, Gray curve – 3 μC cm^-2^.

When the electrode is first placed into solution, it will be at an open circuit potential (OCP) or resting potential. This is the starting potential at *t* = 0 seen in the chronopotentiometry (Figure 2). The OCP varies with electrode and solution properties, but for a mechanically polished electrode in degassed 0.1 M NaCl, it was generally around -50 to -250 mV vs. Ag/AgCl (3 M KCl). Applying a current to the electrode drives electrochemical reactions that changes the electrode potential. A reductive current leads to more negative potentials, an oxidative current to more positive potentials. Increasing the current magnitude results in a larger change in potential.

Applying a -3 μC cm^-2^ pulse for 5 ms in degassed 0.1 M NaCl, the initial potential was -180 mV, and the final potential was -250 mV (Figure 2). The change in potential over time was curved, with decreasing gradient, no plateaus or steps in the curve were seen under any of the conditions tested. On clinical stimulators, a steep rise is seen at the start of the current pulse, called the access voltage, which lasts a few μs (Tykocinski et al., 2005; Mesnildrey et al., 2019), this was not seen on the commercial potentiostat as the minimum sampling time was 10 μs. Increasing the charge density to -10 μC cm^-2^, the initial potential was -220 mV, shifting to -380 mV after 5 ms. Applying positive current pulses, a 3 μC cm^-2^ charge density had an initial potential at -110 mV, ending at -50 mV. And a charge density of 10 μC cm^-2^ started at -70 mV and finished at 70 mV. This demonstrates the larger change in potential seen with higher applied charge densities.

**FIGURE 3:**
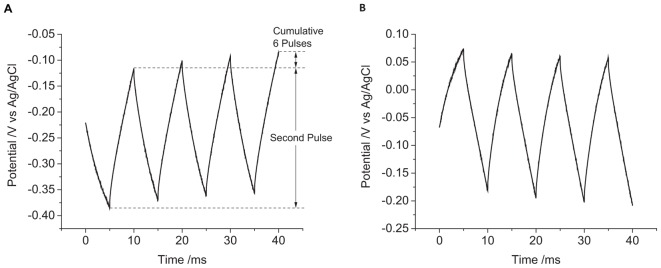
Multiple pulse chronopotentiometry of a mechanically polished 0.6 mm diameter platinum electrode in degassed 0.1 M NaCl. **(A)** Cathodic, **(B)** anodic pulse first with a current density of 10 μC cm^-2^.

The first pulse always displayed a smaller and more variable change in potential than subsequent pulses (Figure 3). To overcome the variability associated with this initial pulse, the charge injection capacity of the electrode was assessed by measuring the change in potential of the second pulse. A summary of the changes in electrode potential under different conditions is given in Table 1.

**Table 1:** Change in potential on a platinum electrode from different chronopotentiometric conditions.

Tested	Electrode Diameter	Polish method	Solution	Degassed	Initial applied charge density (μC cm^-2^)^∗^	Pulse length (ms)	Change in potential (mV)	
	
Variable							Second pulse	Cumulative six pulses
Applied waveform	600 μm	Mechanical	0.1 M NaCl	Yes	–3	5	100 (6)	10 (1)
	600 μm	Mechanical	0.1 M NaCl	Yes	3	5	–98 (5)	–8 (0)
	600 μm	Mechanical	0.1 M NaCl	Yes	–10	5	278 (14)	32 (3)
	600 μm	Mechanical	0.1 M NaCl	Yes	10	5	–257 (1)	–25 (1)
	600 μm	Mechanical	0.1 M NaCl	Yes	–10	10	259 (8)	27 (2)
	600 μm	Mechanical	0.1 M NaCl	Yes	10	10	–241 (8)	–20 (1)
Solution composition	600 μm	Mechanical	PBS	Yes	–10	5	207 (40)	23 (1)
	600 μm	Mechanical	PBS	Yes	10	5	–204 (24)	–19 (5)
	600 μm	Mechanical	Artificial perilymph	Yes	–10	5	220 (14)	27 (4)
	600 μm	Mechanical	Artificial perilymph	Yes	10	5	–219 (22)	–20 (1)
	600 μm	Mechanical	0.1 M NaCl	No	–10	5	270 (10)	27 (3)
	600 μm	Mechanical	0.1 M NaCl	No	10	5	–280 (30)	–21 (5)
Electrode surface	2 mm	Mechanical	0.1 M NaCl	Yes	–10	5	397 (6)	46 (4)
	2 mm	Mechanical	0.1 M NaCl	Yes	10	5	–385 (6)	–36 (4)
	25 μm	Mechanical	0.1 M NaCl	Yes	–10	5	12 (1)	–5 (3)
	25 μm	Mechanical	0.1 M NaCl	Yes	10	5	–14 (0)	–7 (3)
	600 μm	Acid	0.1 M NaCl	Yes	–10	5	420 (15)	43 (2)
	600 μm	Acid	0.1 M NaCl	Yes	10	5	–437 (14)	–39 (2)
	Cochlear implant	–	Artificial perilymph	Yes	–10	5	529 (112)^+^	58 (13)^+^
	Cochlear implant	–	Artificial perilymph	Yes	10	5	–575 (109)^+^	–46 (11)^+^


*^∗^The initial applied charge density is the magnitude of the first current pulse. Subsequent pulses are of opposing polarity at the same magnitude. ^+^Average (standard deviation) of five electrodes.*

When applying a reductive current as the first pulse, the electrode potential becomes more negative than the OCP. The subsequent oxidation pulse then increases the electrode potential above the OCP consistent with a non-ideal electrode. At the conclusion of the eight pulses, the final potential had shifted to more positive potentials than at the end of the previous oxidation pulses. When an oxidation current was applied initially, an opposite change in potential direction was seen. As a result, when using an initial reduction current, the electrode potential obtained more negative potentials then with an initial oxidation current.

**FIGURE 4:**
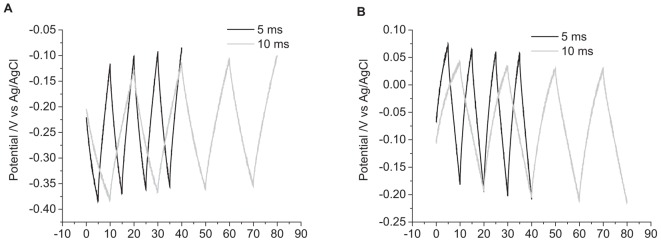
Multiple pulse chronopotentiometry of a mechanically polished 0.6 mm diameter platinum electrode in degassed 0.1 M NaCl. **(A)** Cathodic, **(B)** anodic pulse first with a current density of 10 μC cm^-2^. Black curve – 5 ms pulse, Gray curve – 10 ms pulse.

The current pulse length was set as 5 ms due to the limitations of the potentiostat. To investigate the impact of pulse length on the change in potential, current pulses of 10 ms were applied. To achieve the same charge density of 10 μC cm^-2^, the applied current was also reduced. The impact of changing the pulse length is shown in Figure 4 and in Table 1. Both the change in potential of the second pulse and the cumulative six pulses were smaller when applying longer current pulses.

### Varying Solution Composition

**FIGURE 5:**
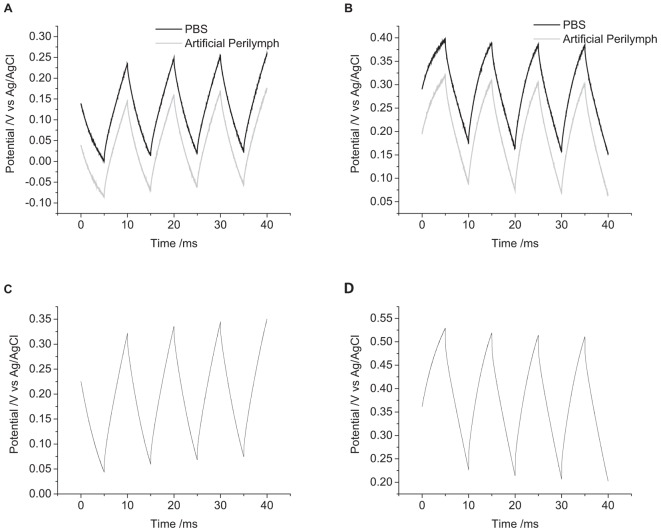
Multiple pulse chronopotentiometry of a mechanically polished 0.6 mm diameter platinum electrode. **(A)** Cathodic, **(B)** anodic pulse first with a current density of 10 μC cm^-2^ in degassed solution. Black curve – PBS, Gray curve – artificial perilymph. **(C)** Cathodic, **(D)** anodic pulse first with a current density of 10 μC cm^-2^ in non-degassed 0.1 M NaCl.

The solution composition was seen to affect the cyclic voltammetric and chronoamperometric response of a platinum electrode (Harris et al., 2018a,b). While electrochemical studies are often undertaken in simple 0.1 M NaCl; cell culture and testing of implantable electrodes are often performed in PBS; while a more accurate model solution for cochlear implants is an artificial perilymph. The OCP of platinum became more positive from 0.1 M NaCl to artificial perilymph to PBS (Figure 5A,B). The change in potential of the second pulse and the cumulative six pulses also decreased from 0.1 M NaCl > artificial perilymph > PBS (Table 1).

The oxygen tension in the body is low, as it is mostly bound to hemoglobin, but it can vary. A higher oxygen concentration was tested by not degassing the solution with nitrogen before performing current pulsing. The OCP of the electrode without degassing was significantly more positive than after degassing (Figure 5C,D). With an initial positive pulse, the change in potential of the second pulse was smaller without degassing, while for an initial negative pulse the change in potential was smaller after degassing (Table 1). After multiple pulsing, the change in potential from the cumulative six pulses was smaller without degassing regardless of the initial potential polarity.

### Varying Electrode Surface

**FIGURE 6:**
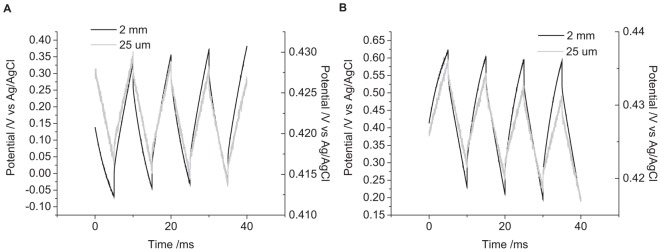
Multiple pulse chronopotentiometry of a mechanically polished platinum electrode in 0.1 M NaCl in degassed 0.1 M NaCl. **(A)** Cathodic, **(B)** anodic pulse first with a current density of 10 μC cm^-2^. Black curve – 2 mm diameter electrode, Gray curve – 25 μm diameter electrode.

There were no trends in OCP with electrode size (Figure 6). However, there was a significant effect on the change in potential with a decrease in magnitude with decreasing electrode size (Table 1).

**FIGURE 7:**
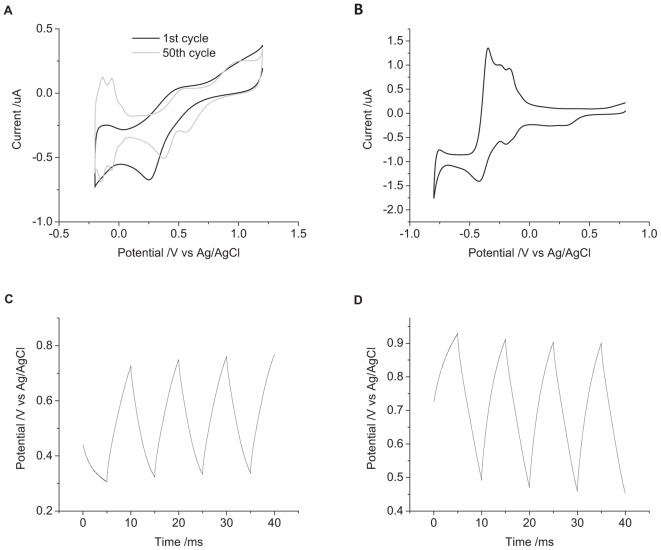
**(A)** First (black) and fiftieth (gray) cycle of a mechanically polished 0.6 mm diameter platinum electrode in 0.5 M H_2_SO_4_ at 50 mV s^-1^. **(B)** First cycle of an acid polished 0.6 mm diameter platinum electrode in degassed 0.1 M NaCl at 100 mV s^-1^. Multiple pulse chronopotentiometry of an acid polished 0.6 mm diameter platinum electrode in degassed 0.1 M NaCl. **(C)** Cathodic, **(D)** anodic pulse first with a current density of 10 μC cm^-2^.

A mechanically polished electrode has a heterogeneous surface with varying levels of oxide and impurities present and a multicrystalline structure. It is possible to clean the electrode surface by cycling the electrode potential in 0.5 M H_2_SO_4_ from 1.2 to -0.2 V at 50 mV s^-1^ for 50 cycles. The voltammetry of an acid-cleaned electrode shows more defined redox peaks associated with oxide formation and hydride adsorption (Figure 7A). Placing the acid-cleaned electrode straight into 0.1 M NaCl then shows a large change in voltammetric response compared to a mechanically polished electrode (Figure 7B). The large reduction peak at -420 mV and oxidation peak at -350 mV are most likely caused by adsorption of chloride to the electrode surface. The chloride adsorption blocks the electrode surface, affecting the Faradaic reactions associated with platinum oxide, hydride, and oxygen (Bagotzky et al., 1970; Li and Lipkowski, 2000; Garcia-Araez et al., 2005). The OCP of the acid-cleaned electrode was significantly more positive than the mechanically polished electrode (Figure 7C,D). The change in potential was also significantly larger for the acid-cleaned than mechanically polished electrode (Table 1). And when applying a 10 μC cm^-2^ anodic first pulse, the electrode potential was raised to 930 mV, well-above the water oxidation potential.

**FIGURE 8:**
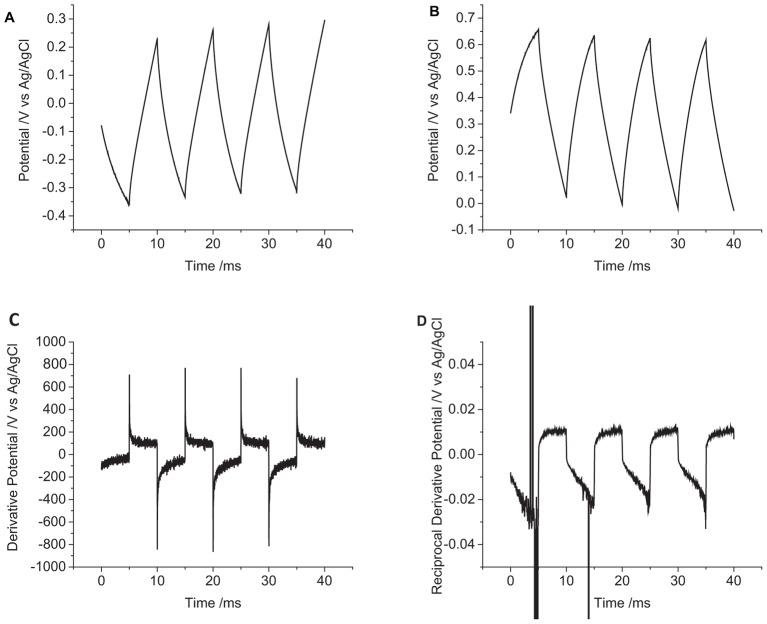
Multiple pulse chronopotentiometry of a non-polished cochlear implant platinum electrode (0.3 mm^2^ nominal area) in degassed artificial perilymph. **(A)** Cathodic, **(B)** anodic pulse first with a current density of 10 μC cm^-2^. **(C)** Derivative and **(D)** reciprocal derivative of curve **(A)**.

The change in potential on the cochlear implant electrode in degassed artificial perilymph was larger than an equivalent sized mechanically polished disk electrode (Figure 8 and Table 1). The relatively large standard deviation from five electrodes is most likely due to variations in electrode area and surface properties.

To aid in visualization of reactions mechanisms occurring during chronopotentiometry, a derivative of the potential/time plot can be performed (Figure 8C). A capacitance current would then appear as a constant; and a Faradaic current would appear as a sharp dip. The reciprocal of the derivative would then transform any Faradaic current into a sharp peak (Figure 8D). The derivative of the cochlear implant chronopotentiometric curve has a larger noise level, but no significant dip in the curve was seen. On the reciprocal derivative curve, the reduction pulses showed an increase over time, while the oxidation current was stable over time; no peaks were seen to indicate a significant variation in the ratio of capacitance and Faradaic current was occurring.

## Discussion

### Effect of Varying Chronopotentiometric Waveform on the Electrode/Tissue Interface

If chronopotentiometric pulsing of platinum in tissue was an ideally polarizable system, only capacitance current would flow. There would be a linear change in potential over time and increasing the current density would increase the rate of potential change (Eq. 1). During biphasic pulsing, an initial reduction pulse would result in a decrease in electrode potential and the following oxidation pulse of the same charge would return the electrode to the initial potential. Conversely, an initial oxidation pulse would raise the electrode potential, the following reduction pulse returning the electrode to the start potential. A different current magnitude (charge density) could be used for the oxidation and reduction pulses, but by adjusting the pulse length, the same charge could still be passed on each. At a truly ideal electrode, there would be no water electrolysis, and so there would be no defined safe potential limits.

At the other extreme, if chronopotentiometric pulsing at the platinum-tissue interface were ideally non-polarizable, only Faradaic current would flow. A reduction pulse would reduce a redox species and an oxidation pulse would oxidize the redox species. Assuming the concentrations of the oxidized and reduced species were minimally altered from the redox reactions, the electrode potential would not change. The use of a reduction or oxidation pulse initially, increasing the current density, pulse length or the inclusion of an interphase gap would have no impact on the electrode potential. An imbalanced charge from oxidation and reduction pulses would also not lead to a shift in electrode potential.

In reality, the platinum-tissue interface is non-ideal. Charge transfer mechanisms will include capacitance and a range of Faradaic reactions including platinum oxide formation and reduction, deposition and stripping of hydride and reduction of molecular oxygen. Capacitance may not be constant with varying electrode potential, and charge available from a Faradaic reaction may have slow kinetics or be exhausted, either by fully oxidizing or reducing the reactant, or due to mass transport limitations. Changes in current magnitude, polarity, number of pulses, and pulse length were all found to affect the change in electrode potential (Figure 2–4 and Table 1). This will be associated with different amounts of each charge transfer reaction occurring. For instance, a decrease in current pulse length would reduce the diffusion time and limit the amount of charge that can be supplied by a solution phase Faradaic reaction (reduction of oxygen). A reciprocal derivative was applied, but due to the complex platinum-solution interface, it was unable to distinguish specific Faradaic reactions (Figure 8). This limits the amount of analysis that can be performed with chronopotentiometry, and so a more general discussion will be made.

The charge transfer mechanisms during a biphasic pulse were affected by initial pulse polarity (Figure 3). For instance, in the presence of a redox active species that can be reversibly reduced, an initial reduction pulse could supply charge by capacitance and the Faradaic reaction (i.e., oxygen reduction and platinum oxide reduction). A subsequent oxidation pulse could also pass capacitance and Faradaic charge. However for an initial oxidation pulse, charge may only be supplied by capacitance. On a subsequent reduction pulse, charge could then be supplied by capacitance and the redox reaction. More complicated reaction mechanisms and mass transport conditions may also affect the oxidation and reduction reactions unequally. As a result, the charge transfer mechanisms from oxidation and reduction pulses may not be the same even with charge balanced pulsing. Multiple electrical pulses may then drive large changes in Faradaic reactions, affecting the concentrations of redox species. This can then shift the electrode potential according to the Nernst equation

(3) $$E=E^{0}-\frac{kT}{nF}\ln\frac{\left[\text{Red}\right]}{\left[\text{Ox}\right]}$$

where *k* is the Boltzmann constant, *T* is the absolute temperature, [Red] and [Ox] are the concentration of the reduced and oxidized species. Indeed, electrode potential changes were seen with just eight pulses (Figure 3) and has been termed ratcheting (Merrill et al., 2005). Ratcheting can lead to large changes in electrode potential, enabling greater platinum dissolution, so changes to the waveform have been attempted to reduce this effect (Kumsa et al., 2016b). This work demonstrates that the ratcheting of the electrode potential can also be controlled by changes to the electrode/solution interface including solution composition (Figure 5), electrode size (Figure 6), and surface structure (Figure 7).

On the cochlear implant (Figure 8), an initial reduction pulse will drive the electrode to more negative potentials. Charge will initially be supplied by capacitance and then platinum oxide and oxygen reduction. If the current density is large enough, hydride adsorption and then water reduction may occur. An oxidation pulse would drive hydride desorption, platinum oxide formation, and capacitance with a final potential higher than the original OCP. For an initial oxidation pulse, the electrode would move to more positive potentials with some platinum oxide formation and capacitance before water oxidation and platinum stripping occurred. And the following reduction pulse would drive capacitance and platinum oxide and oxygen reduction. An initial oxidation pulse may result in greater corrosion of the platinum electrode, but platinum dissolution may also occur through reduction of platinum oxide (Mitsushima et al., 2007).

### Effect of Varying Solution Composition on the Electrode/Tissue Interface

The composition of the solution affected the change in electrode potential (Figure 5 and Table 1). The electrolyte concentration and composition affect the double layer capacitance. Specific adsorption of ions onto the electrode surface can also occur, such as the adsorption of anions onto platinum at positive potentials. Electrochemical studies are often performed in simple electrolytes such as 0.1 M NaCl and cell culture can be performed in PBS. However, these are poor models of the *in vivo* environment, an artificial perilymph displayed an intermediate change in electrode potential at the same charge densities compared to 0.1 M NaCl and PBS.

The oxygen tension in the body is relatively low as it is mostly bound to hemoglobin, but physical activity and changes in the atmosphere do affect the oxygen tension (Misrahy et al., 1958; Tsunoo and Perlman, 1965. Oxygen can be irreversibly reduced at the electrode surface. The initial potential polarity of a biphasic pulse, charge density and oxygen tension will affect the amount of charge supplied by oxygen reduction (Figure 5) (Musa et al., 2011). The presence of other redox active species, including proteins and small organics (e.g., Hemoglobin, dopamine, and amino acids) will also provide sources of Faradaic current. These redox active species will increase the charge injection capacity from a platinum electrode/tissue interface within the water electrolysis window (Donaldson and Donaldson, 1986). An accurate measure of safe charge injection capacity and charge density can only be obtained from an appropriate degassed solution.

### Effect of Varying Electrode Surface on the Electrode/Tissue Interface

The nature of the electrode surface was also found to affect the change in electrode potential (Figure 6, 7). The capacitance of an electrode is dependent on its area. Larger electrodes and increased surface roughness can increase the charge available through capacitance (Tykocinski et al., 2001). For a surface confined redox reaction such as the formation and removal of platinum oxide, a larger electrode surface allows greater capacitance and Faradaic charge. Different crystal planes of platinum also allow a higher density of oxide and hydride adsorption. For a solution phase redox reaction, diffusion of the redox species to the electrode surface will be a planar diffusion profile at short times and on large electrodes. At longer times and smaller electrodes, a radial diffusion profile can be obtained. As a result, a microelectrode can pass a higher charge density before reaching the safe charge injection limit. Assessing the safe charge injection capacity and charge density of an implantable electrode must be made with the clinically relevant roughness, crystal plane, geometry, and size.

A highly clean electrode (planar single crystal with no oxide) would provide less capacitance and Faradaic charge, resulting in a lower charge injection capacity than a rough platinum electrode with oxide present. Here it was seen (Figure 7) that an acid cleaned electrode could enable its potential to rise above the water oxidation potential even though the applied current was well-below the Shannon limit. As implantable electrodes are not highly clean single crystals, an acid cleaned platinum electrode is a poor model for understanding *in vivo* charge transfer mechanisms and safe charge injection capacity. Acid cleaning of implantable platinum electrodes should not be performed before assessing their electrochemical properties.

### Implications for Using Chronopotentiometry at the Electrode/Tissue Interface

Electrochemical analysis of neural electrodes is undertaken to predict the reaction mechanisms that can occur *in vivo* and define the safe charge injection limits and charge densities. The electrochemical methods can involve potential sweeps (cyclic voltammetry) or electrical pulsing (chronoamperometry of chronopotentiometry). Cyclic voltammetry can assist in determining the reaction mechanisms that can occur and their reversibility. However the charge measured from a cyclic voltmmogram must be made from both forward and backward sweeps, and the stability of the electrode under repeated electrical pulses will not be determined (Harris et al., 2018b). Comparison of electrodes must also be made at the same scan rate and over the same potential window. Chronoamperometry can be used to measure the charge passed with a controlled potential pulse, however the charge flux varies over time and this technique is not typically used *in vivo* (Harris et al., 2018a). Chronopotentiometry is the method typically used for stimulating tissue. The limitations of this technique in understanding the reaction mechanisms and defining safe charge density is discussed below. However, correlations can be made between each of these electrochemical techniques.

The safe charge density measured via cyclic voltammetry is obtained by integrating the current-time plot and dividing the charge by an electrode area. The safe charge density was seen to decrease with increased scan rate and increased electrode area; a higher oxygen concentration increased the reduction charge density and decreased the oxidation charge density; the safe charge density increased from 0.1 M NaCl < artificial perilymph < PBS; and acid cleaning increased the safe charge density. In chronopotentiometry, a larger change in potential is due to a lower charge injection capacity. The charge injection capacity measured by chronopotentiometry was similar to cyclic voltammetry, decreasing with shorter pulse length and larger electrode area; a higher oxygen concentration increased the charge injection capacity of an oxidation pulse and decreased the charge injection capacity of a reduction pulse; the charge injection capacity increased from 0.1 M NaCl < artificial perilymph < PBS; but acid cleaning decreased the charge injection capacity. An increase in charge injection capacity also resulted in less ratcheting of electrode potential during multiple pulses. While there are correlations between voltammetric and chronopotentiometric charge injection capacity and charge density, the specific values obtained were different (Harris et al., 2018b).

The chronopotentiometric experiments performed in this article were undertaken in a three-electrode configuration with a well-defined reference electrode isolated from a simple test solution using a commercial potentiostat. When electrical stimulation is performed *in vivo*, a two-electrode configuration is used and the tissue composition is far more complex. In a two-electrode configuration, the platinum reference electrode also functions as the ground electrode. The composition of the platinum/tissue interface is not well-defined and current passing through the reference/ground electrode will drive capacitance and Faradaic reactions, altering the reference/ground electrode potential. As a result, the potential of the platinum/tissue reference/ground electrode is not defined, so it is also difficult defining a safe potential window. In reality, for most cochlear implants, the reference/ground electrode is many times larger than the stimulating electrode so this effect is minimized.

As soon as the electrode array is inserted into tissue, biofouling processes and glial sheath or fibrous tissue encapsulation will begin (Michelson et al., 2018). This will further affect the reference/ground electrode potential and the reaction mechanisms that occur at the electrode. As a result, defining the working electrode potential to a standard potential is difficult, and it will also impact the *in vivo* safe charge injection limits and charge densities. The impact of biofouling and tissue encapsulation are currently not well-represented in *in vitro* studies and this limits the translation of electrochemical results to electrophysiological performance.

The pulse length of neural stimulators is typically around 25 μs with a shorter interphase gap. Most commercial potentiostats have minimum pulse and sample rates in the ms range, preventing measurement of access voltage (μs timescale). Shorter pulse lengths of the same charge density will also drive more capacitance than Faradaic reactions and ratchet the electrode potential further (Figure 4 and Table 1). So while comparison of safe charge density of different electrodes can be made with a commercial potentiostat, they must be tested under the same conditions (e.g., 5 ms pulse length), and great caution must be used when translating this to safe *in vivo* charge densities.

Overall, an accurate measure of safe charge injection capacity, safe charge density and degree of ratcheting must be made with clinically relevant electrodes and conditions. However, this work also demonstrates increased charge injection capacity and reduced ratcheting can be achieved by decreasing the electrode area, reducing the current magnitude, increasing the pulse length or by adding sources of Faradaic current. More detailed studies comparing the electrochemical, electrophysiological and histological response to electrode stimulation are required to define how much of the Shannon plot is due to electrochemical or biological mechanisms (Cogan et al., 2016; Michelson et al., 2018).

### Implications for Impedance Testing of Electrodes

A biphasic chronopotentiometic pulse is also used in an impedance test to provide information on electrode performance. A lower measure from the impedance test should result in lower power usage, *P* = *I*^2^*Z*, and longer battery life. In a uniform conductor, current, and voltage are related through Ohm’s law, *V* = *IR*. In an AC circuit, Ohm’s law must be modified to account for changes in phase, *V* = *IZ*. The impedance is composed of a real and imaginary component (amplitude and phase angle)

(4) $$Z=Z_{real}-jZ_{imaginary}$$

where *j* is used to denote a complex number ($\sqrt{-1}$).

In an electrochemical system, the relationship between current and voltage is complex. The platinum-tissue interface is not a uniform conductor, so Ohm’s law can-not be used to calculate an impedance value from the applied current and measured potential (Tykocinski et al., 2005). This article has demonstrated that variations in chronopotentiometic response may be due to changes in electrode area, capacitance, resistance, topography, and chemical functionality; changes to the surrounding fluid including concentration of redox species and cell encapsulation; electrical noise, movement of the electrodes, or anatomical changes. Many of these factors were seen to affect the potential of a biphasic current pulse (Table 1), so that use of Ohm’s law to calculate an impedance value from the impedance test is not valid. Large variations in response from the impedance test may be caused by insulation failure, electrode shorting or lead wire breakage. Comparisons of impedance over time and across an electrode array may indicate certain mechanisms. However, the complicated nature of the electrode-tissue interface makes it difficult to determine the origins of these effects with just with an impedance test.

## Conclusion

Implantable electrodes stimulate cells with a constant current while the electrode potential changes over time. Charge is supplied by capacitance and Faradaic reactions, the proportions of each depending on conditions at the electrode-tissue interface. In general, a higher charge storage capacity and charge density measured by integrating a cyclic voltammogram results in a smaller change in potential during a chronopotentiometic pulse. Changes to a biphasic pulse waveform (charge density, pulse length and interphase gap) can also affect the charge transfer mechanisms. The charge injection capacity decreased with shorter pulse length and larger electrode area; a higher oxygen concentration increased the charge injection capacity of an oxidation pulse and decreased the charge injection capacity of a reduction pulse; the charge injection capacity increased from 0.1 M NaCl < artificial perilymph < PBS; acid cleaning decreased the charge injection capacity. An increase in charge injection capacity also resulted in a more stable electrode potential after multiple pulses. Understanding charge transfer at an electrode-tissue interface must be obtained from clinically relevant electrodes and conditions (e.g., a cochlear implant in a degassed artificial perilymph). Safe stimulating limits for the same electrode may vary with location in the body. Modification of stimulating method and conditions can be used to increase the charge injection capacity and reduce the ratcheting of an electrode. An impedance test used to assess electrode function is affected by several parameters, and deconvoluting their impact is difficult.

## Author Contributions

AH performed all experimental work. All authors were involved in planning and writing of the manuscript.

## Conflict of Interest Statement

PC was employed by Cochlear, Ltd. The remaining authors declare that the research was conducted in the absence of any commercial or financial relationships that could be construed as a potential conflict of interest.
